# CEM-T4 Cells Do Not Lack an APOBEC3G Cofactor

**DOI:** 10.1371/journal.ppat.1000528

**Published:** 2009-07-31

**Authors:** Guylaine Haché, Reuben S. Harris

**Affiliations:** University of Minnesota, Department of Biochemistry, Molecular Biology and Biophysics, Institute for Molecular Virology, Center for Genome Engineering, Minneapolis, Minnesota, United States of America; The Fox Chase Cancer Center, United States of America

Human APOBEC3G inhibits the replication of Vif-deficient HIV-1 by hypermutating nascent viral cDNA (reviewed in [1]–[3]). Zheng and colleagues recently reported that the HIV-1 restriction activity of APOBEC3G requires a cellular co-factor [4]. Their conclusion depended on three critical observations: i) CEM-T4 cells support the replication of Vif-deficient HIV-1, ii) CEM-T4 cells express restrictive levels of APOBEC3G, and iii) CEM-T4 cells engineered to express more APOBEC3G still permitted Vif-deficient HIV-1 replication [4]. These observations suggested that APOBEC3G alone is insufficient for restriction, and together with subsequent cell fusion experiments, that a recessive cellular co-factor is required. However, the fact that APOBEC3G is capable of restricting a broad number of retroelements, including yeast Ty elements, strongly suggests that other human cellular proteins are not absolutely required for restriction (e.g., [5],[6] and reviewed in [1]–[3]).

**Figure 1:**
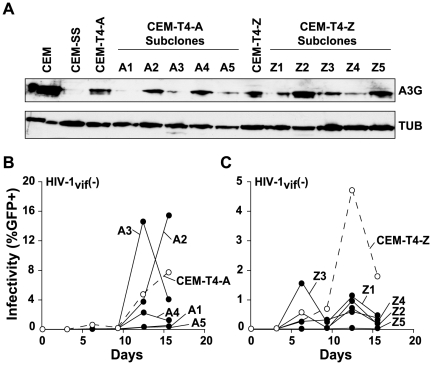
CEM-T4 cells express low APOBEC3G levels and subclones show additional heterogeneity. (A) An immunoblot showing APOBEC3G expression levels in CEM, CEM-SS, CEM-T4, and CEM-T4 subclones. The CEM-T4 line was obtained from both the AIDS Research and Reference Reagent Program (courtesy of Dr. J. P. Jacobs) and Y.-H. Zheng (Michigan State University [4]), designated CEM-T4-A and CEM-T4-Z, respectively. CEM-T4 subclones A1-5 and Z1-5 were obtained by expanding single cells. The anti-APOBEC3G (A3G) antibody was provided by J. Lingappa (University of Washington) through the AIDS Research and Reference Reagent Program, and the anti-tubulin (TUB) antibody was from Covance. (B and C) Replication kinetics of Vif-deficient HIV-1 (IIIB [8],[9]) on the indicated lines or subclones. Virus replication was monitored by mixing cell-free culture supernatant with the reporter cells CEM-GFP, and infectivity was determined by determining the percentage of GFP-positive CEM-GFP cells by flow cytometry [7],[8].

An alternative explanation that could account for the observed permissive phenotype of the CEM-T4 line is that it is mixed, composed of a population of cells expressing low and/or variable levels of APOBEC3G. If this were the case, then the permissive phenotype could simply be due to virus replication in the subset of cells expressing low levels of APOBEC3G. To address this hypothesis, we generated subclones of the CEM-T4 line by serial dilution and determined the level of APOBEC3G expression by immunoblotting (Figure 1A). First, we observed that CEM-T4 cells expressed levels of APOBEC3G that were considerably lower than those in the non-permissive line CEM, regardless of whether they were obtained from the AIDS Research and Reference Reagent Program (CEM-T4-A) or directly from the Zheng laboratory (CEM-T4-Z). Second, we found that representative CEM-T4 subclones, regardless of source, expressed both low and variable APOBEC3G levels. It is further notable that none of the subclones expressed fully non-permissive, CEM-like APOBEC3G levels. This heterogeneity is reflected by the kinetics of Vif-deficient virus replication, with some subclones being fully permissive and others being semi-permissive (Figure 1B and 1C). Nevertheless, these data demonstrated that APOBEC3G levels are both low and variable within individual cells of the CEM-T4 line, thus providing a reasonable molecular explanation for the permissive phenotype of this cell line.

**Figure 2:**
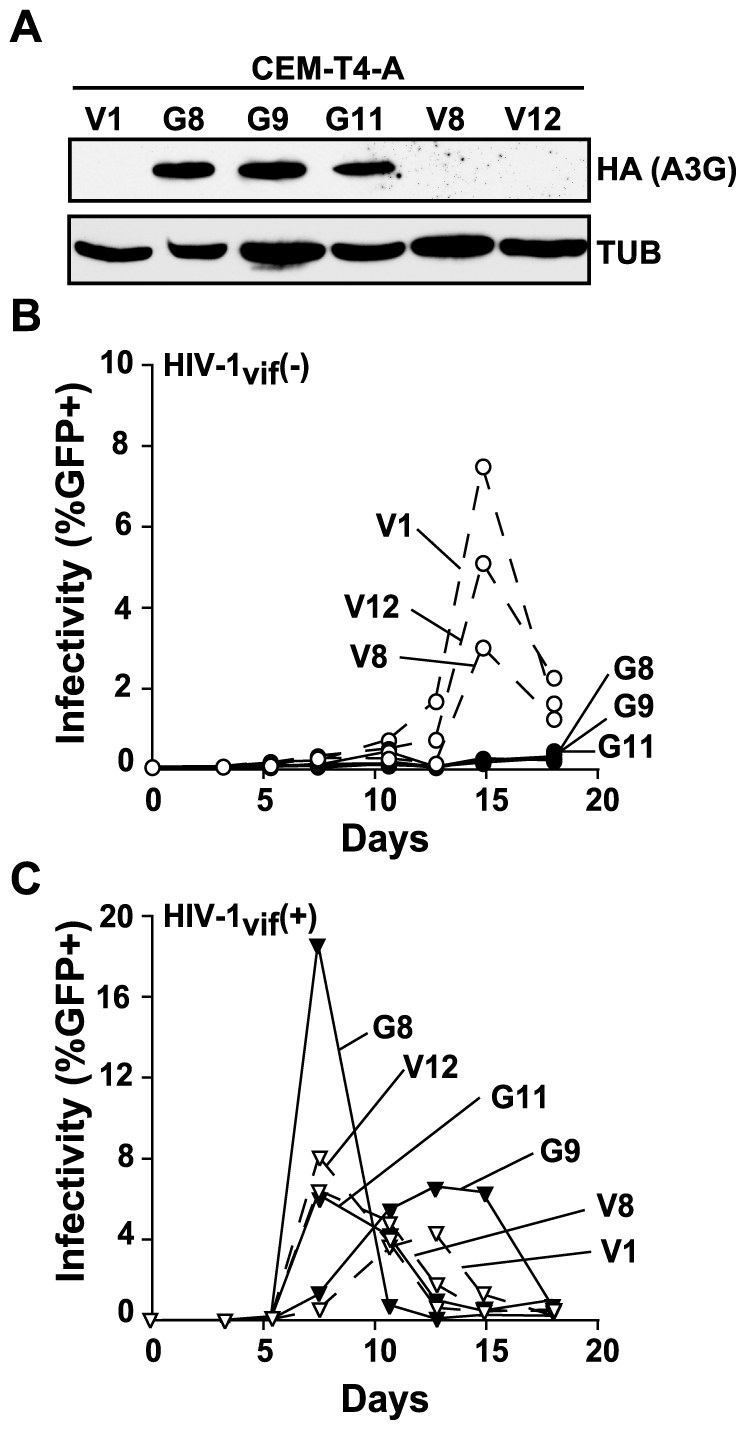
Expression of exogenous APOBEC3G-HA is sufficient to render CEM-T4 cells non-permissive for Vif-deficient HIV-1. (A) An immunoblot showing APOBEC3G-HA expression in CEM-T4 clones G8, G9, and G11 but not in vector control clones V1, V8, and V12. These clones were generated by electroporating an APOBEC3G-HA or vector-control plasmid into CEM-T4-A cells, followed by serial dilution in 96-well plates and outgrowth in selective media containing G418. The anti-HA and anti-tubulin (TUB) antibodies were purchased from Covance. (B and C) Replication kinetics of Vif-deficient or Vif-proficient HIV-1, respectively, on the indicated clones. Solid lines and filled symbols represent virus replication on APOBEC3G-expressing clones and dashed lines and open symbols represent virus replication on vector-control clones.

**Figure 3:**
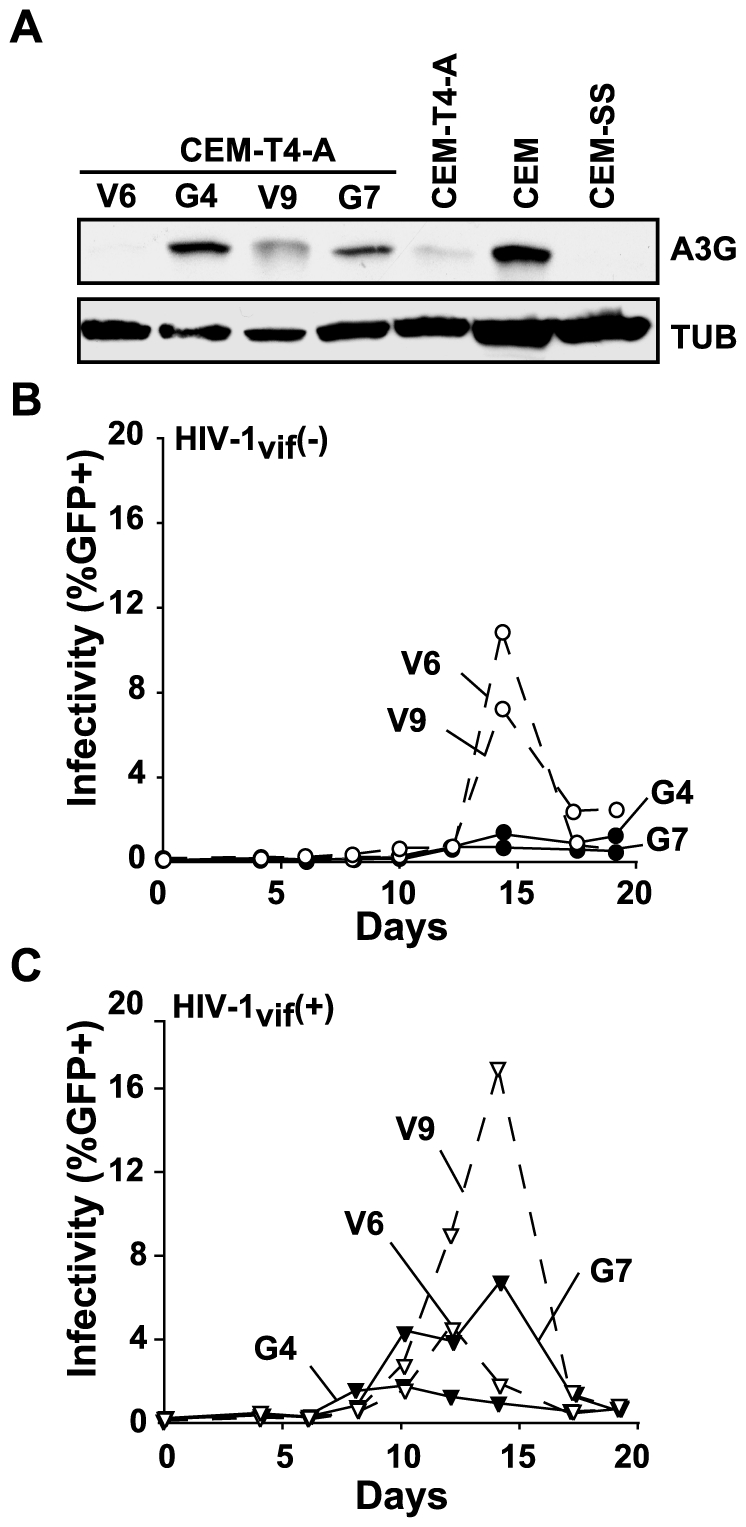
Increasing levels of untagged APOBEC3G is sufficient to render CEM-T4 cells non-permissive for Vif-deficient HIV-1 replication. (A) An immunoblot showing APOBEC3G levels in two individually derived APOBEC3G-expressing CEM-T4 clones (G4 and G7), vector clones (V6 and V9), parental CEM-T4, CEM, and CEM-SS cells. These clones were generated by electroporating an APOBEC3G or vector-control plasmid into CEM-T4-A cells, followed by clone outgrowth in 96-well plates in selective media containing G418. The anti-APOBEC3G (A3G) antibody was provided by J. Lingappa (University of Washington) through the AIDS Research and Reference Reagent Program and the anti-tubulin (TUB) antibody was purchased from Covance. (B and C) Replication kinetics of Vif-deficient or Vif-proficient HIV-1, respectively, on the indicated clones. Solid lines and filled symbols represent virus replication on APOBEC3G-expressing clones and dashed lines and open symbols represent virus replication on vector-control clones.

Curiously, Zheng and coworkers [4] also showed that CEM-T4 cells retrovirally transduced with APOBEC3G were still permissive for Vif-deficient HIV-1 replication. This result may be due to the distinct possibility that APOBEC3G itself inactivated some of the transducing viral cDNAs. Such an attempt at complementation would probably result in a CEM-T4 line that is mixed for APOBEC3G expression. To clarify this important point, we used electroporation to generate panels of HA-tagged and untagged APOBEC3G-expressing CEM-T4 clones and used them for virus replication experiments (Figures 2 and 3, respectively).

Contrary to the findings of Zheng and colleagues [4], our APOBEC3G-expressing CEM-T4 clones were fully restrictive for Vif-deficient HIV-1 replication. The observed non-permissive phenotype was not due to gross overexpression of APOBEC3G because many of the restrictive CEM-T4 clones stably expressed APOBEC3G to levels slightly lower to those of CEM, the original parent of CEM-T4 (e.g., clone G4 in Figure 3). It is also notable that some vector control clones such as V9, like the subclones in Figure 1, expressed low and non-restrictive levels of APOBEC3G. These data suggest that cells must pass an APOBEC3G expression “threshold” before they become fully non-permissive for Vif-deficient HIV-1. Nevertheless, together these virus replication data clearly demonstrated that expression of exogenous APOBEC3G is sufficient to render CEM-T4 cells non-permissive for Vif-deficient HIV-1 replication. We conclude that this cell line does not require a specific endogenous APOBEC3G “co-factor” to restrict Vif-deficient HIV-1.

In this report, we question the obligate APOBEC3G co-factor hypothesis put forth by Zheng and colleagues [4]. Our data clearly provide an alternative explanation that—rather than lacking a co-factor for APOBEC3G—the CEM-T4 line is mixed with individual cells expressing heterogeneous and less-than-restrictive levels of APOBEC3G. Importantly, when transfections were used to generate stable APOBEC3G-expressing CEM-T4 clones, the resulting lines were non-permissive for Vif-deficient HIV-1 replication. Based on these results, we conclude that CEM-T4 cells do not lack an APOBEC3G co-factor and, more generally, that APOBEC3G may not strictly require another cellular protein for HIV-1 restriction. However, we would like to emphasize that neither study rules out the attractive possibility that APOBEC3G, like most other proteins, is likely to be regulated by a variety of cellular processes.
